# The Effect of Ultraviolet Modification on the Performance of Polyamide Fiber-Reinforced Cement Mortars: Optimization and Characterization Using Response Surface Methodology

**DOI:** 10.3390/polym18111358

**Published:** 2026-05-29

**Authors:** Aliye Akarsu Özenç, Ali Mardani, Fatih Özenç, Semiha Eren

**Affiliations:** 1 Textile Engineering Department, Engineering Faculty, Bursa Uludag University, Bursa 16059, Turkey; aakarsu@uludag.edu.tr (A.A.Ö.); semihaeren@uludag.edu.tr (S.E.); 2 Civil Engineering Department, Engineering Faculty, Bursa Uludag University, Bursa 16059, Turkey; fatihozenc@uludag.edu.tr

**Keywords:** polyamide fiber, UV surface modification, cement mortar, mechanical performance

## Abstract

In this study, a UV surface modification method was applied to polyamide (PA) fibers, and its effect on certain fresh and hardened properties of fiber-reinforced cementitious systems was investigated. Within the scope of the study, the individual and interactive effects of fiber volume fraction and UV surface modification time were optimized using the Response Surface Methodology (RSM) based on Central Composite Design (CCD). The optimal performance parameters with CCD were identified at 0.50% fiber content and 18 min of UV exposure. Scanning Electron Microscopy (SEM) and Fourier Transform Infrared Spectroscopy (FTIR) were utilized to analyze the fibers and examine the impacts of surface modification. Three different sample groups were prepared to test the effect of UV treatment after optimization: a control cement mortar without fibers, a polyamide fiber-reinforced mortar without UV treatment, and a polyamide fiber-reinforced mortar with UV surface modification. The tensile, flexural, and compressive strength values of the specimens were determined. The results indicated that UV surface modification led to changes in fiber roughness and an increase in oxygen-containing functional groups on the fiber surface. The data revealed that the mechanical performance of fiber-reinforced composites subjected to surface modification improved (with a 27.4% increase in flexural strength and an 11.3% increase in compressive strength compared to the control samples). The findings indicate that UV surface modification improves the fiber–matrix bond in cement-based systems reinforced with polyamide fibers. UV surface modification emerges as an effective and environmentally friendly method for enhancing the performance properties of fiber-reinforced cement-based systems.

## 1. Introduction

Concrete and mortar, which exhibit high compressive strength and durability, are weak in terms of tensile strength and crack resistance; to mitigate this weakness, fiber reinforcements have become widely used in cement-based composites [1]. The primary function of fiber reinforcement in cement-based mixtures is that the high tensile strength and elastic modulus of the fibers contribute to the overall stiffness and load-bearing capacity of the composite. In addition, the fibers help alleviate stress concentrations at crack tips and enhance the material’s energy dissipation capacity through the crack bridging effect [2,3,4,5]. Fibers utilized in cement-based systems are classified into two categories: synthetic and natural fibers [6]. The choice of fibers for the cement matrix is contingent upon intrinsic qualities, including diameter, density, Young’s modulus, and tensile strength, which directly affect the mechanical performance of the composite [7]. Previous studies have shown that parameters such as fiber length and fiber content play a critical role in determining the final performance of composites [8,9]. However, in addition to these parameters, it is known that the potential contribution of fibers is largely dependent on the effectiveness of the fiber-cement matrix interface.

Synthetic fibers are frequently used to improve the performance of cementitious composites due to their low density, corrosion resistance, and economic advantages [10]. Among synthetic fibers, the use of polyamide fibers is also gaining increasing acceptance [11]. Literature indicates that the flexural and compressive strengths of cement-based systems can be improved by including polyamide fiber reinforcement [12,13,14]. These studies also compared macro and micro polyamide fibers and reported that microfibers generally provide higher compressive and flexural strength and more effective shrinkage control, whereas macrofibers mainly influence post-cracking behavior through different fracture mechanisms. It has also been stated that polyamide fiber addition increases the impact resistance of cementitious systems [15] and enhances resistance to cracking in fire [16].

It is known that the fiber–matrix interface bonding mechanism results from various interaction modes such as physical/chemical bonding, interface separation, plastic material deformations, mechanical bond deformations, and frictional sliding [17]. In addition to interfacial interactions, the long-term durability and dimensional stability of cementitious composites are also critical parameters governing overall performance, and it has been reported that material modifications can significantly influence these properties [18]. Strong interfacial adhesion has been emphasized as a key factor for the full realization of performance improvements [19]. However, despite their superior mechanical properties (such as high tensile strength, toughness, and chemical stability), polyamide fibers cannot provide sufficient interface interaction with cementitious systems due to their hydrophobic surface characteristics [20,21]. The weak adhesion at the fiber–matrix interface limits the crack-bridging capacity of the fibers and prevents the mechanical performance gains from reaching the expected level. Two methods stand out in the literature for improving the fiber–matrix interface bond: the first is based on increasing mechanical interlocking by modifying fiber geometry, and the second is based on modifying fiber surface properties [22].

Several chemical and physical methods have been developed to modify the fiber surface in order to strengthen the fiber–matrix bond [23,24]. Chemical modification methods such as alkali treatments [25], silane coupling agents [26] and coatings [27] strengthen the fiber–matrix bond by creating functional groups on the fiber surface. Although chemical methods provide effective surface changes, they generally have disadvantages such as environmental impacts, high cost, and application complexity [28]. Processes such as plasma [29], ozone [30] and UV are among the physical surface modifications and aim to increase surface roughness and surface energy without leaving residues on the fiber surface.

The increasing emphasis on energy efficiency, waste minimization, and sustainable practices is encouraging research into more environmentally friendly alternative technologies [31]. In this context, ultraviolet (UV) irradiation stands out as an effective and relatively environmentally friendly physical method for modifying polymer fiber surfaces. UV exposure can cause both chemical (photo-oxidation, photochemical reactions) and physical (morphological changes) modifications on the fiber surface, depending on the exposure time and irradiation conditions [32]. While excessive UV exposure can deteriorate polymer fibers, regulated UV irradiation has demonstrated the ability to boost fiber surface polarity and augment adhesion with cementitious matrices, rendering it a viable and eco-friendly approach for interface augmentation [33,34].

Central Composite Design (CCD) is recognized as a powerful experimental design approach widely used within Response Surface Methodology (RSM) [35]. First proposed by Box and Wilson [36] as an efficient alternative to full factorial designs, CCD does not require a three-level full factorial arrangement to estimate a second-order (quadratic) model. This feature allows the determination of nonlinear relationships between independent variables and the response variable. The design, which includes center and axial points in addition to factorial points, is particularly suitable for modeling and optimizing possible curvature on the response surface [37,38]. The following are examples from the literature on fiber-reinforced cementitious systems where the RSM has been used. In addition to statistical approaches such as RSM, machine learning and ensemble learning techniques have also been employed to model and predict cementitious material behavior and optimize process parameters [39].

This methodology facilitates the assessment of interaction effects between UV exposure and fiber content while reducing the number of tests, a feat unattainable by traditional trial-and-error techniques, which are generally time-consuming and resource-intensive as reported in previous studies [40].

This table presents a summary of previous studies from the literature and does not include experimental data from the present work. As shown in Table 1, previous studies have primarily focused on optimizing fiber content or adding additional materials using RSM. The data also reveal that surface modification of fibers, particularly for polyamide fibers, has been relatively less studied. Instead of systematically investigating combined or interactive effects, existing studies have addressed individual parameters. In this regard, the present study distinguishes itself by simultaneously evaluating the combined effect of UV surface modification and fiber volume fraction, thereby providing a more comprehensive understanding of the synergistic effects on the mechanical performance of cement-based composites.

While studies in the literature exist on various chemical and physical surface modification techniques applied to fibers to improve the fiber-cement matrix interface, no studies have been found that specifically examine the fiber-cement matrix interaction following the application of UV modification to polyamide (PA) fibers. Furthermore, current research often investigates the impacts of surface alteration and fiber content separately through conventional trial-and-error approaches. Due to this approach, they are unable to reveal the potential interactions between these parameters. The originality of the study stems from examining these parameters together.

This study aims to address a gap in the literature by investigating the combined effects of UV surface modification and the optimal fiber volume fraction on the mortar performance of PA fiber-reinforced cement composites. The CCD analysis used in this study stands out as a scientific contribution by examining the individual and interactive effects of independent variables and modeling and optimizing these factors, unlike traditional analyses. Using a statistically validated model derived from CCD analysis, the optimal combination of UV exposure time and fiber content was determined, and the fresh and hardened properties of the cement mortars were examined. The results obtained in this study are expected to present UV modification as a feasible, environmentally friendly method for enhancing the performance of synthetic fiber-reinforced cement systems.

## 2. Materials and Methods

### 2.1. Materials

Portland cement of type CEM I 42.5 R, compliant was used as the binding material. The physical, chemical, and mechanical properties of the cement provided by the manufacturer are summarized in Table 2.

Polyamide (nylon 6) fibers with a length of 6 mm were preferred for fiber reinforcement. The basic physical and mechanical properties of the fibers provided by the manufacturer are presented in Table 3.

A water-reducing admixture based on polycarboxylate ether (PCE) with high water-reducing capacity has been used to ensure the workability of the mixtures. The technical properties of the additive, according to the manufacturer’s data, are as follows: density 1.063 g/cm^3^, solid content 32%, pH value 5.8, chloride content less than 0.1%, and alkali content less than 10%.

**Figure 1:**
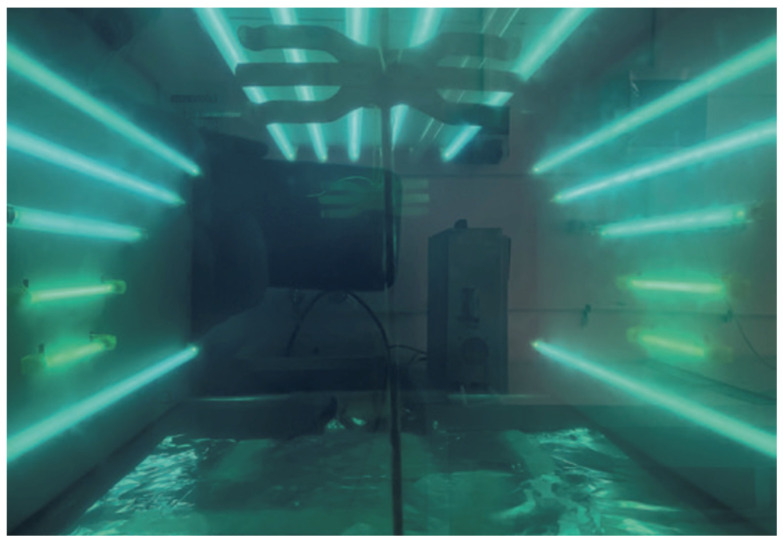
UV cabinet used for fiber surface modification.

The fiber surface modification with UV: The fiber surface modification process was carried out in a specially designed UV chamber (Figure 1). The chamber contains a total of 16 UV-C lamps (wavelength: 254 nm; total power: 470 W) mounted on the top and sides. The dimensions of the specially designed chamber are 100 cm (width) × 70 cm (depth) × 138 cm (height).

During exposure, the fibers were placed at the center of the chamber, approximately 25 cm from the UV lamps (corresponding to the mid-height of the chamber at 35 cm). To guarantee uniform exposure of the fibers to light, they were positioned in a thin, even layer on trays. Due to the lamps situated above and alongside, all fiber surfaces received homogeneous illumination. All irradiation conditions were kept constant throughout the experiments.

### 2.2. Methods

Based on studies in the literature, the UV irradiation times were set at 10, 20, and 30 min. Previous studies have indicated that UV exposure exceeding 30–40 min leads to degradation of the surface of synthetic fibers and a decrease in fiber strength [43]. Therefore, the selected time range was chosen to induce sufficient surface modification without causing damage to the polyamide fibers. However, it has been reported in the literature that the very short irradiation time (e.g., 5 min) is insufficient to observe changes on the surface [44]. In this context, exposure times of 10, 20, and 30 min were determined to achieve effective surface modification without damaging the fiber structure.

#### 2.2.1. Preparation of Mix Mortars and Experimental Plan

A control mortar without fibers and mortar mixtures containing polyamide fibers at volume fractions of 0.25%, 0.50%, and 0.75% were prepared. These volume fractions were selected based on preliminary experiments and previous studies [30], which indicated that this range provides effective mechanical improvement without causing significant workability loss. The same Portland cement (CEM I 42.5 R) was used as the binder in all mixtures. The water/cement ratio was kept constant at 0.50, and the aggregate/cement ratio was maintained at 3.0 (standard sand, according to EN 196-1 [45]). Crushed limestone aggregate with a maximum diameter of 2 mm was utilized as aggregate in the resultant mixes.

**Figure 2:**
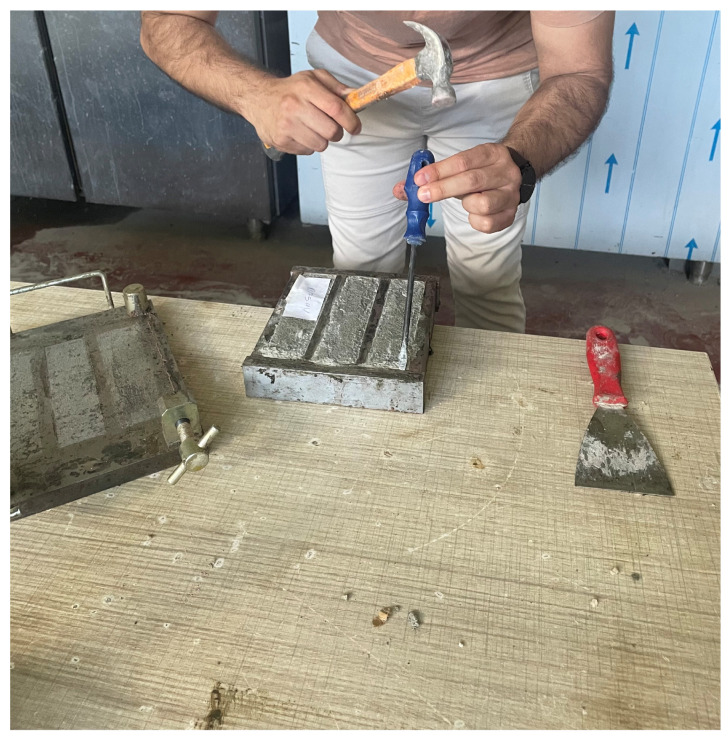
Demolding process of the mortar specimens.

To ensure homogeneous fiber distribution within the mortar matrix, a consistent dispersion method was employed for all mixtures. The fibers were first manually dispersed into a portion of the mixing water to avoid agglomeration. This mixture was then added to the cement and sand during the mixing process, which was performed in a standard mortar mixer according to EN 196-1. The mixing sequence consisted of 30 s at low speed, followed by 30 s of addition of the fiber-water mixture, and finally 60 s at high speed. To determine the compressive strength values, the specimens were placed on 40 mm × 40 mm surfaces. The flexural strengths were determined by conducting a three-point flexural test on prismatic specimens measuring 40 mm × 40 mm × 160 mm. Representative images of the specimens during demolding are presented in Figure 2 to provide a visual reference for specimen preparation and overall integrity. The mixtures were removed from the molds 24 h after production and stored in a lime-saturated water curing tank at a temperature of 23 ± 2 °C until the day of testing.

To achieve consistent workability (target spread diameter of 120 ± 20 mm as per ASTM C1437 [46]) across all mixtures with differing fiber content and UV exposure durations, and to enhance workability, the dosage of the polycarboxylate ether (PCE)-based superplasticizer was modified accordingly. According to information provided by the manufacturer, the chemical additive used in this study has a density of 1.063 g/cm^3^, a solids content of 32%, a pH value of 5.8, a chloride content of less than 0.1%, and an alkali content of less than 10%.

No chemical analysis was performed on the fibers before or after UV modification to quantify surface chemistry changes; however, FTIR analysis was conducted to evaluate possible functional group modifications, and the results are presented in Section 3.2.

Within the scope of this study, CCD was applied within the framework of the Response Surface Methodology (RSM) to determine the optimal fiber content and the optimal surface modification time. It has been emphasized in the literature that flexural strength is one of the tests that best describes the fiber–matrix interface interaction level [47,48,49]. Accordingly, 7-day flexural strength was selected as the response variable. In the initial part of the study, the parameter set yielding the maximum flexural strength within the defined ranges was identified by CCD analysis. In the second stage, final mortar samples were produced using these optimal conditions and subjected to all tests.

#### 2.2.2. Optimization

In optimization studies, 6 mm polyamide fibers were exposed to radiation for specified periods under a UV source with a total power of 470 Watts.

Using a CCD, the effects of fiber mass fraction (A) and UV exposure time (B), as well as their combined effects, on flexural strength were investigated. This design reduced the number of experiments and enabled the estimation of a statistically valid second-order model. This design reliably captures linear, interaction, and quadratic effects while eliminating the need for multiple replicates at each factor level, i.e., the fiber mass and UV exposure [49]. In the CCD design, the central point (0.5% fiber, 20 min UV) was replicated five times, as recommended for the detection of experimental error and reproducibility [49,50]. Independent variables were coded as “Low,” “Medium,” and “High” levels and represented by the values −1, 0, and +1, respectively [49]. The actual (natural) and coded value ranges for each factor are presented in Table 4.

A second-order (quadratic) response model was used to define the relationship between the independent variables and the response variable (flexural strength). The model is expressed by Equation (1), which includes linear, quadratic, and cross-interaction terms:

Y = β_0_ + ∑β_i_x_i_ + ∑β_ii_x_i_^2^ + ∑∑β_ij_x_i_x_j_ + ε (1)

In this equation Y repserent the response variable (flexural strength), β_0_ is the constant term, β_i_ are the linear coefficients, β_ii_ are the quadratic coefficients, β_ij_ are the cross-interaction coefficients, and x_i_ and x_j_ represent the coded independent variables (A and B). ε is the error term that the model cannot explain [51]. The experimental data were fitted to this model, statistically significant terms were determined, and the optimal conditions were estimated.

#### 2.2.3. Tests

The fresh and hardened state properties of all prepared mortars were measured; the results were evaluated comparatively with the fiber-free control sample and the fiber-reinforced samples that did not undergo UV treatment.

As fresh mortar properties, the flow test was performed in accordance with the ASTM C1437 standard to represent workability. The water-reducing admixture dosage that would provide a target flow diameter of 120 ± 20 mm was determined. The fresh unit volume weight was determined according to the ASTM C138 [52] standard.

The mechanical properties of the hardened mortars included flexural and compressive strengths.

Flexural strength tests were performed on prismatic specimens the three-point flexural test specified in the EN 196-1 standard after water curing. Flexural strength values were calculated from the breaking load. The experiments were conducted in three repetitions.

For compressive strength, cube specimens with 50 mm edge length were tested at 7 and 28 days of age after water curing under standard conditions, in accordance with the ASTM C109/C109M standard. The experiments were conducted in three repetitions.

To analyze the effects of surface modifications in detail, Fourier Transform Infrared Spectroscopy (FTIR) and Scanning Electron Microscopy analyses were performed. FTIR analyses were performed on untreated and treated polyamide fibers using a Shimadzu (Kyoto, Japan) device in Attenuated Total Reflectance mode, in the 500–4000 cm^−1^ wavenumber range.

SEM images were acquired using a Zeiss Gemini 300 model Scanning Electron Microscope operating at 10 kV acceleration voltage.

## 3. Results and Discussion

### 3.1. Central Composite Design Analysis

Table 5 presents the test conditions determined using Central Composite Design (CCD)—a method employed to optimize the mechanical performance of mortars produced following UV surface modification of the fibers—and the 7-day flexural strength values obtained under these conditions. The design includes a total of 13 different combinations of two independent variables, namely fiber volume fraction (A) and UV exposure time (B), including center and axial points. The study presents the averages of individual experimental trials. The CCD ensures the statistical reliability of the study by conducting five replicates at the central point (A = 0.5%, B = 20 min) to identify experimental error. Experimental results correspond to individual runs within the RSM design, while repeated center points were used to estimate experimental error and ensure model reliability.

CCD was applied to establish a mathematical model of the relationship between the dependent variable (flexural strength) and the independent variables based on the experimental data obtained. To determine the model type that best reflects the nature of the relationship, the goodness-of-fit statistics for linear, two-factor interactive (2FI), quadratic (second-order), and cubic models were compared (Table 6).

The quadratic model was selected based on its highest R^2^ (0.9709), adjusted R^2^ (0.9501), and predicted R^2^ (0.7778) values, along with the lowest PRESS statistic (4.34) compared to linear, 2FI, and cubic models [53,54]. These metrics indicate that the quadratic model provides the best fit and highest predictive capability for the relationship between fiber content and flexural strength. The F-value shown in Table 7 indicates the ratio of between-group variance to within-group variance. The *p*-value indicates the statistical significance of the model; a value less than 0.05 means the model is significant. df stands for degrees of freedom and refers to the number of independent variables [55]. The model was further validated by Analysis of Variance (ANOVA) (Table 7), indicating the statistical significance of the fiber ratio (A) and its quadratic term (A^2^) (*p* < 0.0001), while non-significant terms (B, AB, B^2^) were subsequently removed to improve model simplicity.

The ANOVA results confirm that the quadratic model is highly significant overall, as indicated by the very low *p*-value (<0.0001) in the “Model” row. Separate examination of the terms shows that the factor with a statistically dominant effect on flexural strength is the fiber volume fraction (A). Both the linear term (A) and the quadratic term (A^2^) of the fiber ratio are highly significant at the *p* < 0.0001 level. This finding provides statistical evidence that the relationship between flexural strength and fiber ratio is not linear but rather a second-order (quadratic) relationship involving a maximum point. In contrast, the *p*-values for the linear and quadratic terms of UV exposure time (B) are 0.3691 and 0.6285, respectively, and are not statistically significant (*p* > 0.05). Similarly, the interaction term between the two factors (AB) is also insignificant (*p* = 0.2082). This result indicates that, within the examined range (10–30 min), the duration of UV exposure does not have a statistically significant independent or interactive effect on flexural strength when compared to the effect of fiber content.

Other important metrics evaluating the reliability of the model and experimental data have also yielded very positive results. The Adequate Precision ratio was calculated as 17.30. The fact that this value is well above the recommended threshold of 4, indicating that the model has an adequate signal-to-noise ratio, demonstrates that the developed model is suitable for making reliable predictions within the design space [54]. The Coefficient of Variation (C.V. %) was low at 2.31%, indicating low experimental scatter and high repeatability of the results. This low value confirms that the experiments were conducted with high precision and minimal random error, reinforcing the robustness of the dataset used for statistical analysis.

By sequentially removing statistically insignificant terms (B, AB, B^2^) from the model and retaining only significant terms (A, A^2^), a simpler and more interpretable final prediction equation was obtained. This model, created using the constant term (β_0_) and significant coefficients, is expressed in Equation (2).

Flexural strength = 13.19 + 1.21 × A − 1.72 × A^2^ (2)

Here, A represents the coded fiber ratio variable (−1: 0.25%, 0: 0.50%, +1: 0.75%). Since the effect of UV duration is not included in the model, the equation defines a relationship valid for a specific UV duration (around 20 min, the center point). The positive linear coefficient (1.21) and negative quadratic coefficient (−1.72) in the equation mathematically express that the flexural strength first increases with the fiber content but then begins to decrease after a certain point. This is the characteristic quadratic behavior of a typical optimization problem.

The overall desirability function of the multi-response system can be estimated by combining the individual desirability function. The desirability function can be expressed as follows: *D* = *d*_1_*^w^*^1^ ⋅ *d*_2_*^w^*^2^ … *d_n_^wn^* where *w_j_* (0 < *w_j_* < 1) represents the weight assigned to the significance of the *j*th response variable *n* and ∑*w_j_* = 1 [56].

Using the second-order model obtained via CCD, the program performed an optimization analysis to achieve the desired response—maximum bending strength. The optimization algorithm recommended a fiber volume fraction of 0.50% and a UV exposure time of 18 min as the solution with the highest “Desirability” value (0.941). The flexural strength value predicted by the model under these optimal conditions is compared with the experimental validation results obtained from specimens prepared under these conditions in Table 8.

**Figure 3:**
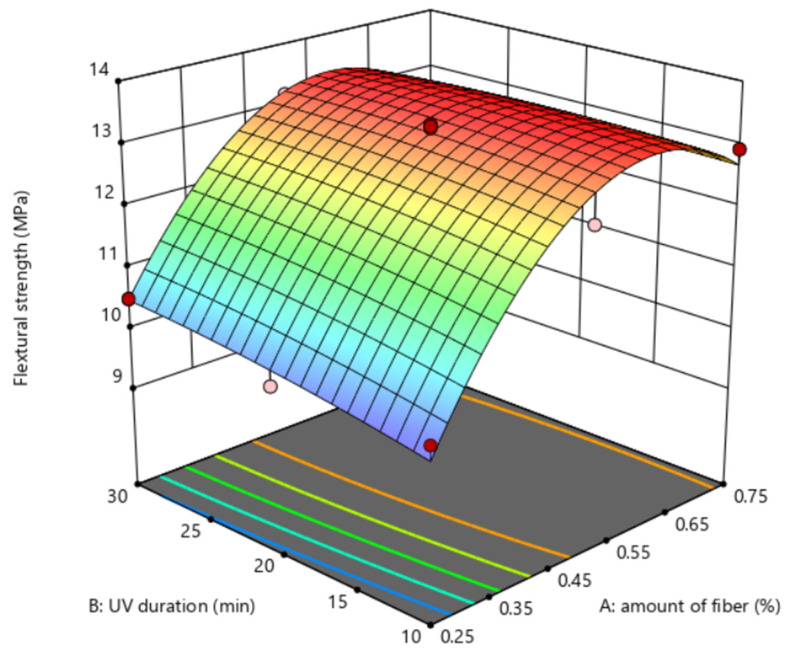
Effects of fiber ratio and UV duration on 7-day flexural strength.

The difference between the predicted (13.32 MPa) and experimental (13.35 MPa) values (less than 0.2%) is negligible. This highlights the predictive accuracy and reliability of the statistical model. It demonstrates that the CCD design is an effective method for determining fiber surface modification and the optimal fiber content. Figure 2 illustrates the effects of fiber surface modification and fiber content on factors influencing flexural strength. Upon examining Figure 3, it is observed that the maximum flexural strength occurs around a fiber content of 0.5% and a UV curing time of 20 min. This finding is consistent with studies in the literature. Previous studies have emphasized that increasing the fiber content up to an optimal level enhances flexural strength by forming an effective bridge against crack propagation and improving load transfer [57,58]. Nevertheless, exceeding this ideal limit (e.g., 0.75%) might detrimentally affect the workability of the mortar matrix, resulting in increased void ratios and complications concerning uniform fiber distribution. This situation can weaken the matrix’s integrity and hinder the expected increase in strength, or even cause a decrease [59,60].

The optimum point observed depending on the UV exposure time indicates the necessity of applying the surface modification process in a balanced manner. Sufficient UV irradiation increases hydrophilicity and roughness on the fiber surface, thereby strengthening fiber–matrix adhesion [27]. However, excessive exposure can cause degradation in the polymer structure and weakening of the fiber’s own mechanical properties, which limits the final performance of the composite.

As a result of numerical optimization, a significant agreement was found between the model prediction and experimental validation under the conditions of a 0.50% fiber ratio and 18 min of UV exposure time (Range Value: 0.941) determined for maximum flexural strength (Table 7). This finding demonstrates that the adopted RSM-CCD methodology is an extremely effective and reliable tool for optimizing the production parameters of UV-modified polyamide fiber-reinforced cement composites. Consequently, the existence of an optimum value for both the fiber reinforcement ratio and the surface modification time indicates that these parameters must be carefully selected to maximize composite performance.

### 3.2. FTIR Analysis Results

**Figure 4:**
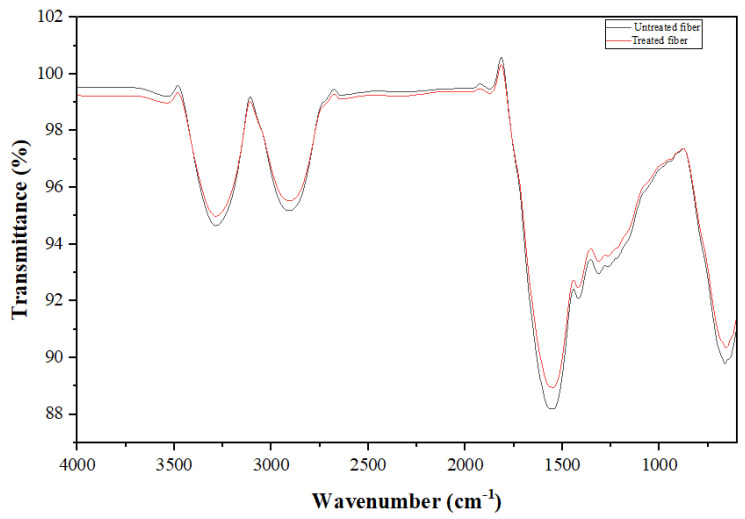
FTIR spectra (overlay) of untreated and UV-modified polyamide fibers. The spectra are offset vertically for clarity. Characteristic bands corresponding to amide I (1630–1640 cm^−1^), amide II (1536 cm^−1^), and N-H stretching (3300–3400 cm^−1^) are indicated.

FTIR analysis was performed on fibers exposed to UV irradiation for 18 min, which was determined to be the optimal surface modification time based on the results of the CCD analysis. Since the maximum flexural strength was determined statistically via CCD analysis, FTIR analysis was not performed on the 10-, 20-, and 30-min samples. Figure 4 presents the results of the FTIR analysis. The analysis revealed that while the backbone structure of the polyamide fibers remained intact, the number of polar functional groups increased following surface modification. This finding appears consistent with the improvement in mechanical properties. Characteristic polyamide bands are clearly observed in both samples. A strong peak around 1630–1640 cm^−1^ originates from the stretching vibration of the carbonyl group (C=O). A broad band in the 3300–3400 cm^−1^ range, corresponding to the N-H stretching vibration known as the Amide I band; and a band around 1536 cm^−1^—known as the Amide II band—resulting from the combination of N-H bending and C-N stretching vibrations, are present [61]. Additionally, C-H stretching vibrations associated with CH_2_ and CH_3_ groups (~2927 and ~3087 cm^−1^) and C-O and C-N stretching vibrations from amide bonds (~1263 and ~1196 cm^−1^) were detected in accordance with the literature [62,63].

It should be noted that the UV treatment did not produce major peak shifts in the FTIR spectra; however, changes in peak intensity and slight band broadening indicate modifications in the surface chemistry of the fibers. This is consistent with UV modification mechanisms reported in the literature, where surface oxidation typically leads to increased concentration of existing functional groups rather than the formation of entirely new chemical bonds [64]. In this study, FTIR analysis was used not so much to quantitatively observe major changes as to examine the trends in the changes in functional groups present in the fiber surface structure following modification.

Following UV modification, two significant changes in the spectrum became apparent despite the preservation of the fundamental polyamide bands. First, a notable increase in the intensity of the Amide I band in the 1630–1640 cm^−1^ region and a slight broadening of the band width were observed. This change indicates an increase in the concentration of functional groups containing carbonyl (C=O) on the fiber surface as a result of photo-oxidative reactions caused by UV-C irradiation. Secondly, a shift in intensity was observed in the band around 1500 cm^−1^ reflecting changes in the N-H bending and C-N stretching vibrations; this band is associated with the Amide II region and results from the rearrangement of hydrogen bonds due to UV-induced surface oxidation. As previously mentioned, UV surface modification was performed under normal laboratory atmospheric conditions. The primary reactive species responsible for the resulting surface oxidation are molecular oxygen (O_2_) and oxygen-derived reactive species formed during UV irradiation, namely oxygen radicals. Through these reactive species, photo-oxidative reactions are initiated on the polyamide surface, leading to the formation of functional groups containing additional carbonyl groups.

The combined evaluation of these spectral findings indicates that UV radiation triggers oxidative functionalization on the surface of polyamide fibers. This process may involve the removal of hydrogen from polymer chains and their subsequent reaction with atmospheric oxygen, leading to the formation of new carbonyl groups (aldehydes, ketones, or carboxylic acid derivatives). The results obtained are in full agreement with literature studies reporting that UV treatment improves the hydrophilicity of polyamide-based fibers and polymers by increasing their surface energy [65]. In conclusion, FTIR analysis proves that UV modification successfully achieves a surface chemical change that can critically affect the interaction with the cement matrix. It is expected that the increase in oxygen-containing functional groups and the resulting change in surface polarity will enhance the fiber-cement matrix interaction. This study did not measure interactions at the interface in detail; however, prior research indicates that oxygen-containing functional groups on polymer surfaces can interact with Ca^2+^ ions and silanol groups present in cement hydration products like calcium silicate hydrate (C-S-H) [30]. Such interactions could contribute to improved fiber–matrix adhesion and the resulting increase in mechanical performance, which will be discussed in the following section.

### 3.3. SEM Analysis Results

**Figure 5:**
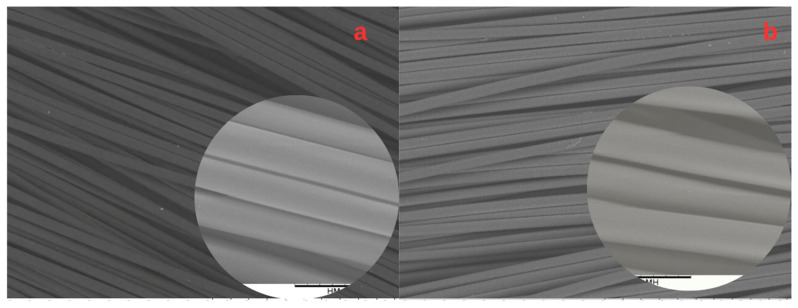
SEM images of untreated (**a**) and UV-modified (**b**) polyamide fibers at different magnifications (500× and 800×). Scale bars represent 20 μm.

The physical effects of UV modification on the surface morphology of polyamide fibers were examined by SEM, and representative images of untreated and treated fibers are presented in Figure 5. The observations highlight the potential changes that may occur following UV surface modification through a qualitative analysis.

Micrographs in Figure 4 reveal that the overall structure of the fiber is preserved after UV treatment and that the surface modification does not cause significant damage to the fiber surface. This indicates that optimal UV surface modification does not damage the fiber structure. It is observed that fiber homogeneity is slightly reduced after the UV treatment. This may be ascribed to the formation of micro-scale fissures subsequent to surface modification; however, greater magnification imaging is necessary for verification. The SEM images are consistent with the FTIR analysis results, and it is reasonable to conclude that UV irradiation may increase surface roughness, which in turn could enhance mechanical interlocking at the fiber–matrix interface [27]. This synergistic mechanism constitutes one of the fundamental reasons for the notable improvements in flexural and compressive strength discussed in the following section.

### 3.4. Performance Evaluation of Optimized Mixtures

#### 3.4.1. Fresh-State Properties

**Figure 6:**
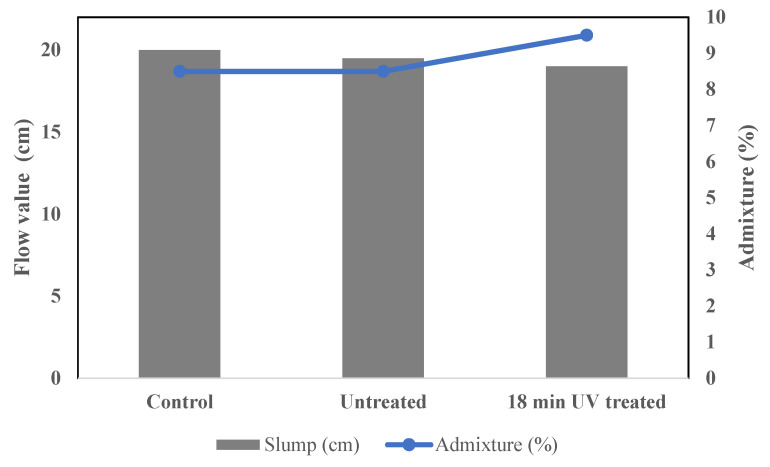
Comparative evaluation of flow diameter and superplasticizer dosage of the specimens.

The fresh-state properties of the specimens prepared under optimum conditions (0.50% fiber, 18 min UV treatment) are presented in Figure 6 together with the fiber-free control mixture and the mixture containing untreated fibers for comparison. The spread diameter, which is the primary indicator of workability, was measured as 20 cm and 19.5 cm for the control and untreated fiber-reinforced mixtures, respectively, at similar admixture dosages. The percentages of the admixture shown in Figure 6 were calculated based on the weight of the cement. The observed differences in flow values are relatively small and should be interpreted as indicative trends rather than statistically significant variations.

However, a different behavior was observed in mixtures incorporating UV-modified fibers. In these mixtures, a slight increase in the required dosage of superplasticizer was observed in order to achieve the same target flow value (120 ± 20 mm). This behavior is directly associated with the morphological changes induced on the fiber surface by UV modification. As emphasized in Section 3.3, it was noted that fiber homogeneity changes somewhat following UV treatment and that this phenomenon is associated with microscale roughness. The increase in surface roughness increases inter-fiber friction and aggregate/mortar interactions, thereby increasing flow resistance and necessitating the use of more additives to maintain the desired workability. The literature highlights that an increase in surface roughness has a negative effect on mixture rheology [27]. The results obtained indicate that fiber surface modification not only affects the properties of the hardened state but also influences the properties of the fresh state, significantly altering the mixture’s rheological behavior.

#### 3.4.2. Flexural Strength Results and Analysis of Mechanisms

**Figure 7:**
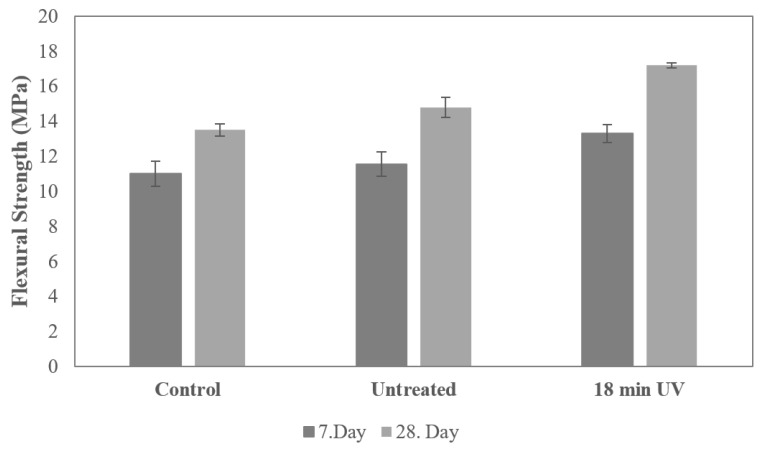
Comparative graph of the 7- and 28-day flexural strength values of different specimen groups.

The flexural strength performance of the control, untreated, and UV-cured composites at 7 and 28 days is shown in Figure 7. The statistical analysis of the samples was interpreted within the framework of CCD, and the ANOVA results are presented in Section 3.1. Upon examining Figure 6, it is observed that the addition of fibers to the cement matrix increases the composite’s flexural strength. This increase in strength can be explained by the crack bridging mechanism of the fibers. Fibers prevent the propagation of microcracks and transfer stress along the crack surfaces, thereby increasing the material’s fracture toughness and ultimate load-bearing capacity [66,67,68,69].

When the flexural strengths at 28 days were examined, it was found that adding fibers to the mixtures resulted in a 9.6% increase in flexural strength compared to the control sample. This increase was observed more distinctly in fiber-reinforced composites with surface modification. Samples treated with UV surface modification exhibited approximately 15% higher strength on day 7 compared to the control sample, and this difference became more pronounced by day 28, reaching 17.2 Mpa, which corresponds to a 27.4% increase compared to the control sample and a 16.2% improvement compared to the sample reinforced with untreated fibers.

The increase in flexural strength can be attributed to the physical and chemical changes induced by UV surface modification on the fiber surface. According to FTIR analysis results, the amount of oxygen-containing functional groups on the UV-exposed fiber surface increases [70]; this is expected to enhance surface hydrophilicity and wettability [71]. This situation may promote better physicochemical interaction with the cement matrix. Additionally, the fact that the fibers appear less homogeneous in SEM analysis after UV treatment—a phenomenon associated with microcrack formation—suggests that an increase in surface roughness, which could contribute to mechanical interlocking, may be occurring. Although specific chemical interactions at the interface were not directly investigated in this study, literature studies have reported that oxygen-containing functional groups on polymer surfaces can interact with Ca^2+^ ions and silanol groups in the C-S-H phases, and that this could potentially improve fiber–matrix adhesion [23,30].

These findings are fully consistent with the literature reporting that UV pretreatment strengthens fiber–matrix adhesion in various polymer and composite systems. For example, improvements in interfacial bonding and flexural properties after UV pretreatment have been reported in glass fiber composites [72] and jute/glass hybrid composites [73]. In cementitious systems, UV-treated polymer particles have also shown improved compatibility with the matrix, leading to enhanced tensile behavior [74].

In conclusion, UV surface modification induces both chemical and physical transformations in polyamide fibers, fundamentally strengthening their interfacial interaction with the cement matrix. The improvement in the fiber–matrix interface has led to an increase in flexural strength by enabling better load transfer through the fibers. The results revealed that the UV irradiation time, as determined by CCD analysis, facilitated surface functionalization and increased roughness without causing elongation in the fiber structure. The increase in flexural strength can be attributed to the superior crack bridging capability resulting from improved interfacial bonding and the enhanced energy dissipation at the fiber–matrix interface.

#### 3.4.3. Compressive Strength Behavior and the Matrix-Strengthening Effect of UV Modification

**Figure 8:**
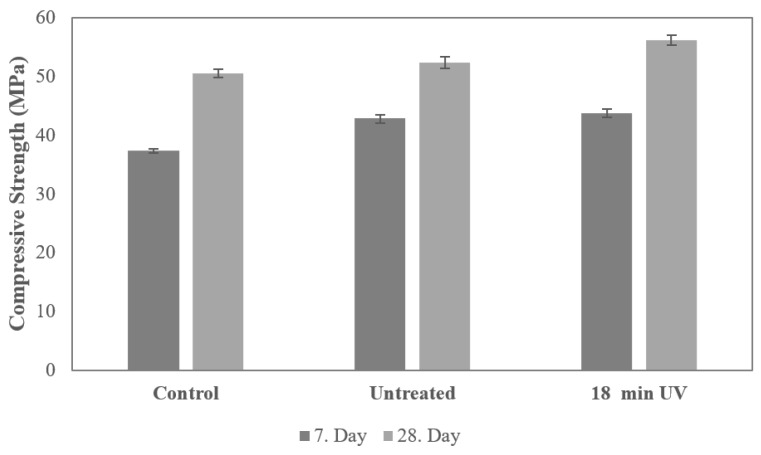
Comparative graph of the 7- and 28-day compressive strength values of different specimen groups.

The compressive strength performance of the control, untreated, and UV-cured composites at 7 and 28 days is shown in Figure 7. The statistical analysis of the samples was interpreted within the framework of CCD, and the ANOVA results are presented in Section 3.1. Upon examining Figure 8, it is observed that the addition of fibers to the cement matrix increases the composite’s compressive strength.

As expected, the results of the study showed an increase in strength in all samples during the transition from 7 days to 28 days. However, as is well known, the contribution of fiber addition to compressive strength is generally lower than its effect on flexural strength. The 28-day compressive strength of the control sample was determined to be 50.5 MPa. When the 28-day compressive strengths of the specimens were compared, it was found that the untreated polyamide fiber-reinforced specimens exhibited a 3.8% increase in compressive strength compared to the control specimens, reaching 52.4 MPa. This modest effect can be attributed to the coalescence of microcracks and the delay in macrocrack formation (the crack-bridging effect of the fibers).

The 28-day compressive strength of the UV-modified fiber-reinforced composites was recorded as 56.2 MPa. When compared to the other specimens, this result corresponds to an 11.3% increase relative to the control specimen and a 7.3% increase relative to the untreated fiber-reinforced composites.

The underlying mechanism of this performance enhancement is closely related to the interfacial improvement mechanisms discussed in the flexural strength section. As confirmed by FTIR and SEM analyses, UV modification induced both chemical (increased polarity and hydrophilicity) and physical (enhanced surface roughness) transformations on the fiber surface. Under compressive loading, when microcracks developing within the matrix encounter a fiber, the improved interface may contribute to performance through several mechanisms described in the literature [5,75]:

First, crack tip blocking and deflection: A strongly bonded fiber acts as a barrier along the crack propagation path. The crack must expend additional energy to overcome this obstacle and typically propagates by deflecting around the fiber or changing direction. This process slows crack growth and dissipates energy [5].

Second, mitigation of the triaxial stress state: Fibers may help reduce stress concentrations within the matrix and distribute the load over a wider area, potentially alleviating the triaxial stress condition that develops under compression and delaying brittle failure [73].

Although the specific contributions of these mechanisms were not directly measured in this study, it is reasonable to infer that the interface strengthened by UV modification may enhance their efficiency. Based on the FTIR and SEM findings, improved chemical adhesion and increased surface roughness could promote better load transfer and crack resistance, consistent with observations reported in the literature [23,76]. Consequently, these combined effects may contribute to the higher compressive strengths observed for UV-modified fiber-reinforced specimens.

Previous studies have reported that UV surface modification alters the hydrophobic properties of synthetic fibers, improves fiber–matrix compatibility, and has a positive effect on mechanical properties [23]. For example, a study conducted by Aliyu et al. [77] reported that UV surface modification improved the compressive strength of cement-based systems reinforced with polypropylene fibers. These results are consistent with the findings of the present study and underscore that UV surface modification may be an effective method for enhancing compressive strength.

## 4. Conclusions

The results obtained from CCD analysis showed that the greatest increase in flexural strength occurred with a 0.5% fiber content and 18 min of UV exposure. FTIR analysis results showed an increase in oxygen-containing functional groups on the fiber surface. SEM analysis revealed that fiber homogeneity was slightly lower in the UV-treated sample compared to the untreated sample.

When examining the properties of the fresh state, it was determined that the processability of the mixtures decreased due to increased roughness on the fiber surface following UV treatment. Increases in flexural strength (a 27.4% increase compared to the control samples) and compressive strength (an 11.3% increase compared to the control samples) were observed in UV-modified fiber-reinforced composites. Considering the improvement in the mechanical properties of cement-based systems resulting from UV surface modification applied to fibers, this method’s low cost, ease of application, and eco-friendly nature may make its industrial use feasible in the future.

## Figures and Tables

**Table 1:** Summary of selected literature studies employing Response Surface Methodology (RSM) in fiber-reinforced cementitious composites.

Author(s) & Year	Fiber Type	Surface Modification Method	Optimization/Statistical Method	Optimized Parameters	Target Properties
Algahifi et al. [41]	Polypropylene	carbon nanotube	RSM	Fiber and carbon nanotube content	Compression, tensile, flexural and impact strength
Gong et al. [42]	Basalt	Rubber	RSM	Water/binder ratio, basalt fiber and rubber content	Compression and flexure strength
Şahin et al. [29]	Carbon	Ozon	RSM	Fiber content and ozone treatment time	Workability, flexural and compressive strength

**Table 2:** The physical, chemical, and some mechanical properties of cement.

Oksit (%)	%	
SiO_2_	18.00	
Al_2_O_3_	4.75	
Fe_2_O_3_	3.58	
CaO	63.00	
MgO	1.40	
Na_2_O + 0.658 K_2_O	0.70	
SO_3_	3.11	
Specific gravity		3.06
Specific surface (cm^2^/g)	3441	
Compressive Strength (MPa)	7-day	42.8
	28-day	51.8
Setting time (min)	Initial	170
	Final	240

**Table 3:** Some physical and mechanical properties of polyamide fibers.

Specific Gravity	Fiber Length (mm)	Tensile Strength (GPa)	Modulus of Elasticity (GPa)	Melting Temperature (°C)
1.14	6	0.8–1.1	3.0–3.5	260

**Table 4:** Independent variables and their levels used in the optimization study.

Factor	Unit	Lower Limit (−1)	Middle Point (0)	Upper Limit (+1)
A-Fiber mass fraction	%	0.25	0.50	0.75
B-UV exposure time	minute	10	20	30

**Table 5:** CCD experimental matrix and measured 7-day flexural strength values.

Experimental	A: Fiber Ratio (%)	B: UV Time (min)	Flexural Strength (MPa)
1	0.5	20	13.26
2	0.25	10	10.10
3	0.5	20	13.29
4	0.75	30	12.54
5	0.5	20	13.27
6	0.75	20	12.40
7	0.5	10	12.5
8	0.5	30	13.16
9	0.5	20	13.33
10	0.75	10	12.93
11	0.5	20	13.31
12	0.25	20	10.01
13	0.25	30	10.5

**Table 6:** Design Models.

Source	*p*-Value	R^2^	Adjusted R^2^	Predicted R^2^	Press
Linear	0.0480	0.4551	0.3461	0.0044	19.44
2FI	0.7228	0.4631	0.2841	−0.8165	35.43
Quadratic	<0.0001	0.9709	0.9501	0.7778	4.34
Cubic	0.4833	0.9782	0.9478	−1.5076	48.95

**Table 7:** Results of analysis of variance (ANOVA) for the quadratic model.

Source	Sum of Squares	df	Square Mean	F Value	*p* Value
Model	18.95	5	3.79	46.68	<0.0001
A: Fiber ratio	8.81	1	8.81	108.49	<0.0001
B: UV time	0.0748	1	0.0748	0.9215	0.3691
AB	0.1560	1	0.1560	1.01	0.2082
A^2^	8.14	1	8.14	100.25	<0.0001
B^2^	0.0208	1	0.0208	0.2558	0.6285

Model Statistics: R^2^ = 0.9709, Adjusted R^2^ = 0.9501, Adeq. Precision = 17.30; C.V. % = 2.31.

**Table 8:** Prediction of optimum conditions and experimental verification results.

Fiber Ratio (%)	UV Time (min)	Desirability	Predicted Strength (MPa)	Experimental Strength (MPa)
0.50	18	0.941	13.32	13.35

## Data Availability

The data presented in this study are available on reasonable request from the corresponding author. The data are not publicly available due to ongoing research activities.
